# Adherence to Inflammatory Bowel Disease Medications in Southern New Zealand

**DOI:** 10.1093/crocol/otab056

**Published:** 2021-08-02

**Authors:** Kristina Aluzaite, Rhiannon Braund, Liam Seeley, Obreniokibo Ibifubara Amiesimaka, Michael Schultz

**Affiliations:** 1 Gastroenterology Research Unit, Department of Medicine, DSM, University of Otago, Dunedin, New Zealand; 2 Department of Preventive and Social Medicine, New Zealand Pharmacovigilance Centre, Dunedin School of Medicine, University of Otago, Dunedin, New Zealand; 3 Gastroenterology Unit, Dunedin Hospital, Southern District Health Board, Dunedin, New Zealand

**Keywords:** medication adherence, inflammatory bowel diseases, IBD, Otago, New Zealand

## Abstract

**Background:**

Inflammatory bowel diseases (IBDs) require continuous clinical management; poor medication adherence may result in worse disease outcomes and increased healthcare costs. This study investigated medication adherence and associated risk factors in IBD patients.

**Methods:**

Otago (New Zealand) IBD patients were mailed questionnaires on demographics, medication-taking behavior, and a validated Probabilistic Medication Adherence Scale (ProMAS).

**Results:**

The response rate was 29.7% (*n* = 174/590). The study sample was mean (SD) 50.5 (16.9) years old, 57.9% female, 49.4% had Crohn’s disease, and 43.9% ulcerative colitis, with median of 9.5 years (interquartile range: 5.0–22.0) of IBD duration. About 31.1% scored below medium adherence according to ProMAS. About 11.9%, 24.7%, and 23.1% reported failing to renew, purposely not taking, and stopping taking medications, respectively; 27.2% of those who reported having no issues taking medication scored below medium on the ProMAS. Older age was associated with higher ProMAS adherence score (Pearson’s *r* = .25; *P* = .0014). There were no differences in medication adherence between the types of IBDs (*P* = .87), disease activity status (*P* = .70), or gender (*P* = .27). There was no correlation between the number of medications and level of adherence (Pearson’s *r* = .09; *P* = .27). About 18.7%, 10.1%, and 5.0% of patients reported forgetting to take medications when traveling, when out of routine, and when busy, respectively. The most used strategies to remember medications included utilizing specific routines (40.1%) and keeping medications in specific locations (21.1%).

**Conclusions:**

A third of IBD patients had below medium medication adherence. There were discrepancies between self-reported and tool-assessed medication adherence scores with over one-third of patients underestimating/overestimating their adherence.

## Introduction

Inflammatory bowel diseases (IBDs) are complex chronic conditions with the 2 main types being Crohn’s disease (CD) and ulcerative colitis (UC).^1^ Inflammatory bowel disease cases are diagnosed mainly in late adolescence or early adulthood years, resulting in a lifelong burden of medication and disease monitoring.^2^

Inflammatory bowel disease medications are taken to induce and/or maintain remission, and include regular oral or rectal medications, subcutaneous injections, and regular infusions. Disease management also involves lifestyle changes, such as dietary modifications and regular endoscopic examinations.^3, 4^ The combinations of treatments that IBD patients receive are heterogeneous, ie, patients might be prescribed several medications with different dosing regimens, which may make disease self-management more challenging and distressing.^5^

Studying patients’ medication adherence is challenging due to the complex and multifactorial nature of the concept with varying anticipated effects on disease outcomes. Nonadherence is defined as a deviation from the prescribed treatment regimen, but there is a lack of consensus on specific cutoffs for adequate adherence, with a frequently used arbitrary threshold of >80% being considered indicative of good medication adherence (this varies with the route of administration).^6, 7^ Overall, poor medication adherence is associated with worse symptoms and disease outcomes, such as formation of abscesses, or stricturing disease, which then may lead to surgery, more hospitalizations, and consequently increased healthcare costs.^8–10^

A number of studies have reported low levels of medication adherence in IBD patients utilizing varying adherence definitions and different tools used to measure it.^7^ The medication nonadherence estimates vary between patient groups and by the type of medications they use, with values like 12.1%-13.3%^11^ to 45%^10, 12^ for mesalazine, 9% to 32% for immunomodulators,^7^ and 22.1% to 54.3% for infliximab^13, 14^ reported. These estimates are likely affected by the study participant selection criteria, study design, and the definition of adherence and hence are difficult to generalize.

As a result, monitoring medication adherence is challenging.^7^ The most accurate method would be direct observation or testing biological samples for drug or metabolite levels (therapeutic drug monitoring); however, this is not feasible in real life for most medications taken at home. Therapeutic drug monitoring may also often be problematic as the available accuracy varies between medications, and many factors may affect the drug metabolite levels.^15, 16^ Tablet counts, pharmacy refill data, and electronic technologies, such as Medication Event Monitoring System (MEMS) caps for drug bottles, are some of the other examples to measure medication adherence. However, these are expensive, time-consuming, or pose specific patient population challenges. Medication Event Monitoring System caps, while promising in their concept, still do not guarantee medication adherence and at this point lack evidence for efficacy in the *real-world* setting.^7^ Finally, a number of self-assessment questionnaires have been developed to capture the complexity of medication-taking behavior, such as the Medication Adherence Report Scale (MARS) instrument,^17^ and the 8-item Morisky Medication Adherence Scale^18^; the latter was found to have a poor correlation with medication adherence as indicated by the pharmacy refill data.^19, 20^ The Probabilistic Medication Adherence Scale (ProMAS) questionnaire was more recently developed and is better able to discriminate between patients’ adherence levels than the MARS.^21, 22^ Further research has shown that ProMAS results are closer to those obtained via other objective assessments and it covers a wider range of medication-taking behaviors than the MARS-5 survey.^23^ As the ProMAS was also freely available, it was chosen for this study.

Several factors have been linked with poor medication adherence in IBD patients, such as younger age at diagnosis, activity of disease, and feelings of depression or anxiety.^11^ Likewise, the most common self-reported reasons for poor medication adherence include forgetfulness and being away from home.^24^ Also, IBD patients frequently employ strategies to facilitate their medication adherence including keeping medications at specific locations and using pill containers.^25^

The goal of this study was to investigate the medication-taking behaviors of IBD patients in Otago, New Zealand (NZ), and compare self-recognized medication adherence challenges with a more formal medication adherence measure. We also aimed to identify any risk factors associated with worse adherence to IBD medications to inform future interventions and clinical practice.

## Materials and Methods

### Participant Recruitment

Southern District Health Board (SDHB) patients, residing in Otago, NZ, above 15 years old, and known to have IBD (local IBD-specific database EpiSoft, Sydney, Australia), were invited to participate in the study via post. Patients who consented to the study were mailed questionnaire packages that included questions on demographics, disease-related information, medication-taking behavior, and a validated ProMAS.^21^ Participants completed the questionnaires independently and returned them in the provided pre-paid envelop. Eligible participants were contacted a second time in cases where they did not return their completed questionnaires within 3 weeks post initial contact time.

### Questionnaire Scoring

The medication adherence questionnaire consisted of 2 parts: 18 questions designed by the research team to identify possible reasons for nonadherence and the ProMAS. Probabilistic Medication Adherence Scale was scored as per Kleppe et al.,^21^, with the following combined scores indicating low adherence (0–4), moderate-low adherence,^5–9^ moderate-high adherence,^10–14^ and high adherence.^15–18^ The questionnaires were scored using TeleForm (OpenText Corp., Canada; version 11.2) and manually curated.

### Data Analysis

Descriptive statistics for the data were derived, with missing data excluded from each individual factor statistic (number of responses specified next to each statistic). Comparisons between participant groups and associated factors were done using *t*-tests and Pearson correlations, respectively. *P*-values at or below .05 were considered statistically significant. Analysis was performed using R statistical computing language.^26^

### Ethical Considerations

This study received the University of Otago Ethics Committee approval (H17/083) that complies with the Declaration of Helsinki standards. Study participants signed written informed consent forms.

## Results

### Study Sample

The study response rate was 29.5% (*n* = 174/590). Furthermore, 10 participants were removed due to low questionnaire completion, resulting in 164 responses being included in the analysis.

The study sample was mean (SD) 50.5 (16.9) years old, 57.9% (*n* = 95/164) female, 89.6% (*n* = 147/164) NZ European, and 3.0% (*n* = 5/164) Māori.

About 49.4% (*n* = 81/164) of the study participants had CD, 43.9% (*n* = 72/164) UC, and 3.7% (*n* = 6/164) had IBD unspecified with median IBD duration of 9.5 years (interquartile range: 5.0–22.0). About 63.1% (*n* = 101/160) of the respondents had active disease, and 87.2% (*n* = 143/164) were taking medications for IBD; 8% (*n* = 12/150) reported not taking any medications, 68.7% (*n* = 103/150) took 1–2 medications, 16.7% (*n* = 25/150) took 3–5 medications, and 6.7% (*n* = 10/150) took more than 5 medications. The most used IBD medications were 5-aminosalicylic acid (5ASA)-containing medications (54.2%), immunomodulators (39.6%), biologic agents (18.1%), and corticosteroids (8.3%) (Supplementary Data Content 1). While data on medication frequency and dose were collected, the response error rate appeared to be high, and hence these data were not included in the analysis.

### Medication Adherence

About 4.9% (*n* = 8/164), 26.2% (*n* = 43/164), 41.5% (*n* = 68/164), and 27.4% (*n* = 45/164) scored low, medium-low, medium-high, and high on ProMAS questionnaire, respectively; 11.9% (*n* = 19/160), 24.7% (*n* = 39/158), and 23.1% (*n* = 37/160) reported failing to renew, purposely not taking, and stopped taking medications, respectively.

**Figure 1.**
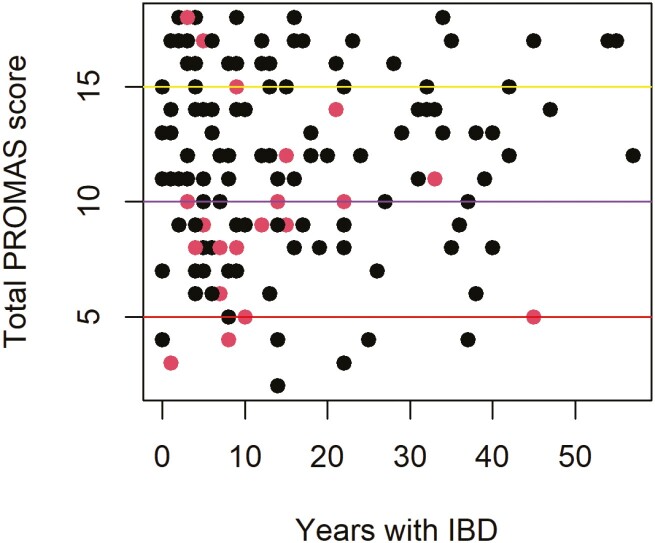
Perceived trouble taking medications vs ProMAS medication adherence scores*. *Red points indicate participants who reported having trouble taking medication and black points—no trouble; red line indicates low adherence, purple—low-medium adherence, and yellow—high adherence. Abbreviation: ProMAS, Probabilistic Medication Adherence Scale.

About 27.2% (*n* = 37/136) of the participants who reported having no issues taking medication scored low to low-medium on the ProMAS questionnaire, while 50% (*n* = 12/24) of those who reported having issues had above medium adherence, as indicated by ProMAS scores (Figure 1). Participants who reported having issues taking medications scored significantly lower in the ProMAS questionnaire (*P* = .036). These patients listed switching healthcare facilities (34.8%, *n* = 8/23), being in remission (17.4%, *n* = 4/23), administration of medicine (13.0%, *n* = 3/23), and relationship with the doctor (8.7%, *n* = 2/23) as the main reasons for having trouble taking medications.

### Factors Associated With Levels of Medication Adherence

**Figure 2.**
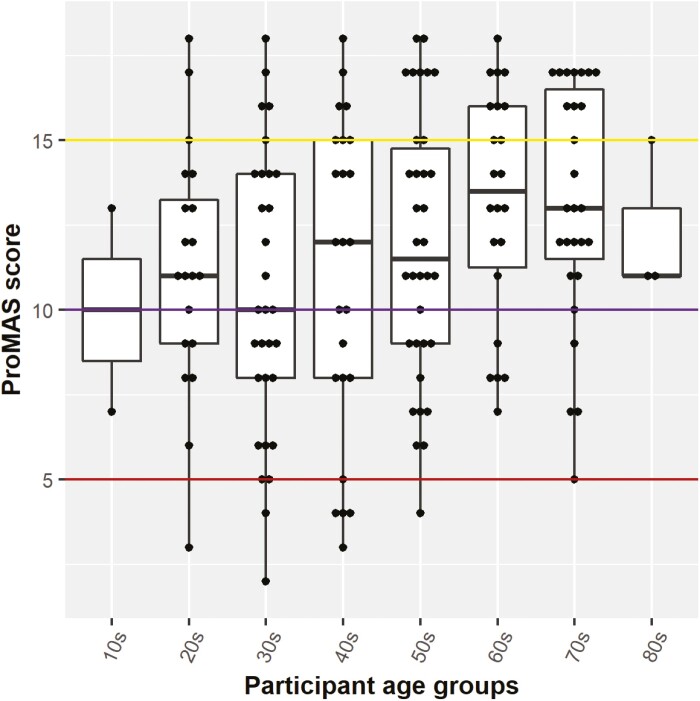
Medication adherence scores (ProMAS) by participant age groups*. *The boxplots show median, 25th and 75th percentile of scores per age group along with distribution of all the scores. Each point represents one participant. Red line indicates low adherence, purple – low-medium adherence, yellow – high adherence. Abbreviation: ProMAS, Probabilistic Medication Adherence.

There was a weak positive correlation between participants’ age and ProMAS adherence score (Pearson’s *r* = .25; *P* = .0014; Figure 2). This indicates that with increasing age, there is increased adherence.

There was no significant difference in medication adherence between the types of IBD (*P* = .87), disease activity status (*P* = .70), and gender (*P* = .27). There was no correlation either between the number of medications used (Pearson’s *r* = .07; *P* = .37) or the duration of IBD (Pearson’s *r* = .03; *P* = .70) and medication adherence.

### Barriers to Medication Adherence

About 36.9% (*n* = 60/164) of the study participants listed specific circumstances for not adhering to their medications, with 18.7%, 10.1%, 5.0%, and 4.3% of them reported forgetting to take medications when traveling or away, when out of routine, when busy or in a hurry, and when going out in the evening, respectively; 56.8% (*n* = 79/139) of the participants specifically stated there never being any circumstances, where they would forget to take their medications. There was strong evidence that patients, who listed specific circumstances for nonadherence, had lower adherence scores, compared with patients who stated there being no circumstances for nonadherence (*P* < .0001) (Supplementary Data Content 2).

About 12.2% (*n* = 20/164) of the study participants listed reasons for failing to renew their medications that included not noticing when the medications ran out (20%), “not wanting to” renew medications (10%), side effects (10%), and not being able to afford their prescriptions (10%); 22.6% (*n* = 37/164) of the patients reported not taking their medications on purpose, 27.0% of them due to other medical/health reasons, 16.2% to “test what would happen,” and 13.5% due to side effects.

### Strategies for Medication Adherence

About 92.7% (*n* = 152/164) of the participants listed specific strategies (free text) to improve their medication adherence (Table 1). The most used strategies to remember medications included having specific timing or adhering to a specific routine (40.1%), keeping medications in specific locations (21.1%), and using medication-planning containers (9.2%).

**Table 1.** Strategies and barriers to medication adherence: qualitative responses.

Question	% (*n*)
How do you remember to take your medications?	Total (*n* = 152)
• Use specific timing/routine	**40.1 (61)**
• Keep medications in a specific location	21.1 (32)
• Use a pill planner	9.2 (14)
• Reminder phrase	5.9 (9)
• Use a phone/email reminder	5.3 (8)
• Keep medications in a visible location	5.3 (8)
• Use a calendar	3.3 (5)
• Take together with other medications	1.3 (2)
• No problems remembering	8.6 (13)
Are there particular circumstances where you are more likely not to take medications?	Total (*n* = 139)
• None	**56.8 (79)**
• Traveling/being away from home	**18.7 (26)**
• Change of routine	10.1 (14)
• Going out in the evening	5.0 (7)
• When busy/ in a hurry	4.3 (6)
• On Holidays	3.6 (5)
• Intentional	3.6 (5)
• When tired	0.7 (1)
Failed to renew prescription medication due to:	Total (*n* = 20)
• Did not notice when medications finished	**20 (4)**
• Side effects	10 (2)
• Intentional	10 (2)
• Cannot afford prescription	10 (2)
Purposely not take medications due to:	Total (*n* = 37)
• Due to other medical/health reasons	**27.0 (10)**
• Testing what would happen	16.2 (6)
• Side effects	13.5 (5)
• Did not want to	13.5 (5)
• No IBD symptoms	5.4 (2)
• Pills are too big	5.4 (2)
• Hunger (meal-medication timing)	2.7 (1)
• Did not improve the symptoms	2.7 (1)
Purposely stopped taking medications for:	Total (*n* = 26)
• Less than a week	19.2 (5)
• 1 week to 1 month	**23.1 (6)**
• Less than 6 months	**23.1 (6)**
• More than 6 months	15.4 (4)
• Dose reduction	19.2 (5)

Abbreviation: IBD, inflammatory bowel diseases.

## Discussion

This study investigated IBD patient medication adherence and associated barriers in SDHB patients, living in Otago, NZ, and identified discrepancies between the perceived medication adherence and a more objective ProMAS assessment, alongside considerable overall levels of poor medication adherence. Otago-based SDHB IBD patients were mailed questionnaires, with 29.7% returning them, constituting a response rate similar to those of several studies adopting this “cold” survey approach in assessing IBD patients, reporting rates of 7.6%-31.7%.^27–29^

Studies show that poor medication adherence can result in worse disease outcomes, more hospitalizations, and patients’ worse quality of life. Given the chronic and unpredictable nature of IBD and the need for careful ongoing disease management, investigating medication adherence is essential to optimize disease management. According to Trindade et al.,^30^ 77% of American IBD physicians screened for medication adherence, but only 19% of the 77% used formal screening measures, such as pill counts, prescription refill rates, or adherence surveys, highlighting that the problem of medication nonadherence is not adequately identified or addressed even in clinical settings.

Our study found that 31.1% of surveyed IBD patients in Otago, NZ, had below medium adherence, which is in line with other studies conducted on medication adherence in IBD patients.^29, 31^ Specific comparisons between medication nonadherence levels in IBD patients are challenging due to the variability in medication adherence measures used, anticipated medication-specific differences, and lack of a consensus on nonadherence thresholds. Case in point, Selinger et al.^32^ found more aggressive therapies to be associated with higher medication adherence (5-aminosalicylates, with the lowest adherence, followed by thiopurines, with biological therapies having the highest adherence), but this may also be due to the route of medication administration. While some studies show nonadherence increasing with the frequency of medication intake,^33^ a recent Cochrane review found no difference in oral 5ASA treatment adherence among different dose regimens.^34^ Furthermore, large in-depth studies and consensus guidelines for adequate medication adherence cutoffs are required to facilitate research and clinical care of IBD patients.

Studies show that information-seeking behavior and perceived disease control result in higher medication adherence and better disease outcomes.^11, 35^ Hence, in this study, we sought to compare self-reported perceived medication adherence with a more objective ProMAS estimate. About 27.2% of the participants who reported being adherent to their medications had below medium adherence as assessed via the ProMAS. Moreover, half of the patients who indicated having adherence issues had above medium adherence using the ProMAS. These findings suggest that 1 in 3 patients’ self-evaluation of their medication adherence would not align with the assessments from a less-biased standardized tool. This highlights the importance of using formalized and comprehensive adherence monitoring tools, eg, validated medication adherence questionnaires, particularly in clinical settings. However, it is noteworthy that patients who explicitly stated that there were no circumstances where they would miss their medications had significantly higher medication adherence.

A number of studies identified a list of factors associated with poor medication adherence with mixed evidence for each.^7^ While higher disease knowledge has been linked with improved disease management strategies and psychological variables, several studies found no relationship between patients’ IBD knowledge levels and perceived medication adherence.^36, 37^ However, low medication-associated knowledge and inability to recall this information were linked with worse medication adherence in IBD patients.^8, 11, 38^ There is mixed evidence for the use of knowledge-based interventions to improve medication adherence in IBD patients; with Tiao et al.^39^ showing a significant improvement in long-term medication adherence after a pharmacist-led adherence counseling intervention, whereas Waters et al.^40^ did not find a statistically significant difference. Patients-specific personal characteristics and perceptions, such as information-seeking behaviour^35^ and higher perceived disease control,^11^ have been linked with higher medication adherence.

We found older age to be correlated with increased medication adherence, emphasizing the need to communicate the importance of medication adherence for younger patients, as they might be dealing with the lifelong consequences of their poor adherence. We did not find any adherence differences between the genders or types of IBD, which is consistent with most studies.^7, 32, 41, 42^ Surprisingly, we did not find any correlation between either duration of disease or number of medications and medication adherence. However, similar results have been reported in several studies, indicating that other factors play a more important role in medication adherence.^42^

About 92.7% of the study participants listed specific strategies to improve their medication adherence with the most common methods being incorporating taking medications into their daily routine (40.1%) and keeping the medications in specific locations (21.1%), which are commonly reported strategies in similar studies.^5^ Also, 23.1% of the patients purposely stopped taking their medication, some of which wanted to test what would happen or due to side effects. This should be addressed with better education strategies to improve patients’ understanding of the nature of the disease. While there is mixed evidence on the efficacy of educational interventions in improving medication adherence,^43^ higher perceived disease control has been linked with positive outcomes. Hence, future studies should focus on identifying specific perceptions and barriers that result in poor medication adherence and design appropriate interventions to address these.

The main limitation of this study is the self-selecting nature of the investigation, likely capturing only the most adherent patients. The opposite is also possible, with patients who would be aware of their medication-taking behavior challenges being interested in participating in the study. Second, this was a retrospective study; hence, all responses are subject to a recall bias. Third, study participants completed the questionnaire independently, without any supervision, which left room for possible misinterpretations of the questions; while the questionnaires were designed to be reader friendly, reading and language ability may have affected the study responses. This was an anonymous study; hence, we could not link disease outcomes and specific medication use profiles with the medication adherence. However, we anticipate that the anonymous nature of the study would have resulted in more honest responses. Further in-depth studies are required to identify and define the specific scope of the problem to design intervention strategies.

## Conclusions

We identified important discrepancies between self-perceived medication adherence and a more objective assessment. In plain terms, patients both overestimate and underestimate their medication adherence levels. This is relevant to the clinical environment, supporting the need for a formal measure of medication adherence. We also confirmed substantial levels of low medication adherence among IBD patients in SDHB and identified high levels of patients who were willing to experiment with their treatment regime, which highlights the need for better communication strategies between the patients and clinicians.

## Supplementary Material

otab056_suppl_Supplementary_Appendix

## Data Availability

Data are not publicly available.

## References

[1] Baumgart DC , Carding SR. Inflammatory bowel disease: cause and immunobiology. Lancet. 2007;369(9573):1627–1640. 17499605 10.1016/S0140-6736(07)60750-8

[2] Loftus EV Jr . Clinical epidemiology of inflammatory bowel disease: incidence, prevalence, and environmental influences. Gastroenterology. 2004;126(6):1504–1517. 15168363 10.1053/j.gastro.2004.01.063

[3] Harbord M , Eliakim R, Bettenworth D, et al. Third European Evidence-based Consensus on Diagnosis and Management of Ulcerative Colitis. Part 2: Current management. J Crohns Colitis. 2017;11(7):769–784. doi:10.1093/ecco-jcc/jjx009 28513805 10.1093/ecco-jcc/jjx009

[4] Gomollón F , Dignass A, Annese V, et al; ECCO. 3rd European Evidence-based Consensus on the Diagnosis and Management of Crohn’s Disease 2016: Part 1: Diagnosis and medical management. J Crohns Colitis. 2017;11(1):3–25. 27660341 10.1093/ecco-jcc/jjw168

[5] Plevinsky JM , Greenley RN, Fishman LN. Self-management in patients with inflammatory bowel disease: strategies, outcomes, and integration into clinical care. Clin Exp Gastroenterol. 2016;9:259–267. 27601930 10.2147/CEG.S106302 PMC5003515

[6] Sabaté E. Adherence to Long-Term Therapies: Evidence for Action. World Health Organization Report; 2003.

[7] Lenti MV , Selinger CP. Medication non-adherence in adult patients affected by inflammatory bowel disease: a critical review and update of the determining factors, consequences and possible interventions. Expert Rev Gastroenterol Hepatol. 2017;11(3): 215–226. 28099821 10.1080/17474124.2017.1284587

[8] Tae CH , Jung SA, Moon HS, et al Importance of patients’ knowledge of their prescribed medication in improving treatment adherence in inflammatory bowel disease. J Clin Gastroenterol. 2016;50(2):157–162. 26501880 10.1097/MCG.0000000000000431

[9] Kane S , Huo D, Aikens J, Hanauer S. Medication nonadherence and the outcomes of patients with quiescent ulcerative colitis. Am J Med. 2003;114(1):39–43. 12543288 10.1016/s0002-9343(02)01383-9

[10] Kawakami A , Tanaka M, Nishigaki M, et al Relationship between non-adherence to aminosalicylate medication and the risk of clinical relapse among Japanese patients with ulcerative colitis in clinical remission: a prospective cohort study. J Gastroenterol. 2013;48(9):1006–1015. 23208019 10.1007/s00535-012-0721-x

[11] Severs M , Mangen MJ, Fidder HH, et al Clinical predictors of future nonadherence in inflammatory bowel disease. Inflamm Bowel Dis. 2017;23(9):1568–1576. 28700534 10.1097/MIB.0000000000001201

[12] Ribaldone DG , Vernero M, Saracco GM, et al The adherence to the therapy in inflammatory bowel disease: beyond the number of the tablets. Scand J Gastroenterol. 2018;53(2):141–146. 29228844 10.1080/00365521.2017.1405070

[13] Martelli L , Lopez A, Strobel S, et al Adherence to infliximab therapy in inflammatory bowel disease patients in a real-life setting. J Dig Dis. 2017;18(10):566–573. 28858439 10.1111/1751-2980.12539

[14] Ma C , Evaschesen CJ, Gracias G, et al Inflammatory bowel disease patients are frequently nonadherent to scheduled induction and maintenance infliximab therapy: a Canadian cohort study. Can J Gastroenterol Hepatol. 2015;29(6):309–314. 26069894 10.1155/2015/378628 PMC4578454

[15] Cramer JA , Scheyer RD, Mattson RH. Compliance declines between clinic visits. Arch Intern Med. 1990;150(7):1509–1510. 2369248

[16] Chan W , Chen A, Tiao D, et al Medication adherence in inflammatory bowel disease. Intest Res. 2017;15(4):434–445. 29142511 10.5217/ir.2017.15.4.434 PMC5683974

[17] Horne R , Weinman J. Patients’ beliefs about prescribed medicines and their role in adherence to treatment in chronic physical illness. J Psychosom Res. 1999;47(6):555–567. 10661603 10.1016/s0022-3999(99)00057-4

[18] Tan X , Patel I, and Chang J. Review of the four item Morisky Medication Adherence Scale (MMAS-4) and eight item Morisky Medication Adherence Scale (MMAS-8). Innov Pharm. 2014;5(3): 165.

[19] Kane S , Becker B, Harmsen WS, et al Use of a screening tool to determine nonadherent behavior in inflammatory bowel disease. Am J Gastroenterol. 2012;107(2):154–160. 22306937 10.1038/ajg.2011.317

[20] de Castro ML , Sanromán L, Martín A, et al Assessing medication adherence in inflammatory bowel diseases. A comparison between a self-administered scale and a pharmacy refill index. Rev Esp Enferm Dig. 2017;109(8):542–551. 28679280 10.17235/reed.2017.5137/2017

[21] Kleppe M , Lacroix J, Ham J, Midden C. The development of the ProMAS: a Probabilistic Medication Adherence Scale. Patient Prefer Adherence. 2015;9:355–367. 25784791 10.2147/PPA.S76749 PMC4356448

[22] Lam WY , Fresco P. Medication adherence measures: an overview. Biomed Res Int. 2015;2015:217047. 26539470 10.1155/2015/217047 PMC4619779

[23] Vluggen S , Hoving C, Schaper NC, De Vries H. Psychological predictors of adherence to oral hypoglycaemic agents: an application of the ProMAS questionnaire. Psychol Health. 2020;35(4):387–404. 31588778 10.1080/08870446.2019.1672873

[24] Ingerski LM , Baldassano RN, Denson LA, Hommel KA. Barriers to oral medication adherence for adolescents with inflammatory bowel disease. J Pediatr Psychol. 2010;35(6):683–691. 19776229 10.1093/jpepsy/jsp085 PMC2902844

[25] Kawakami A , Tanaka M, Naganuma M, et al What strategies do ulcerative colitis patients employ to facilitate adherence? Patient Prefer Adherence. 2017;11:157–163. 28203059 10.2147/PPA.S117841 PMC5293502

[26] Team RDC. R: A language and environment for statistical computing. R Foundation for Statistical Computing; 2008. http://www.R-project.org.

[27] Rubin DT , Siegel CA, Kane SV, et al Impact of ulcerative colitis from patients’ and physicians’ perspectives: results from the UC: NORMAL survey. Inflamm Bowel Dis. 2009;15(4):581–588. 19067414 10.1002/ibd.20793

[28] Nahon S , Lahmek P, Saas C, et al Socioeconomic and psychological factors associated with nonadherence to treatment in inflammatory bowel disease patients: results of the ISSEO survey. Inflamm Bowel Dis. 2011;17(6):1270–1276. 21560190 10.1002/ibd.21482

[29] Horne R , Parham R, Driscoll R, Robinson A. Patients’ attitudes to medicines and adherence to maintenance treatment in inflammatory bowel disease. Inflamm Bowel Dis. 2009;15(6): 837–844. 19107771 10.1002/ibd.20846

[30] Trindade AJ , Morisky DE, Ehrlich AC, et al Current practice and perception of screening for medication adherence in inflammatory bowel disease. J Clin Gastroenterol. 2011;45(10):878–882. 21555953 10.1097/MCG.0b013e3182192207 PMC3156931

[31] van der Have M , Oldenburg B, Kaptein AA, et al Non-adherence to anti-TNF therapy is associated with illness perceptions and clinical outcomes in outpatients with inflammatory bowel disease: results from a prospective multicentre study. J Crohns Colitis. 2016;10(5):549–555. 26738757 10.1093/ecco-jcc/jjw002 PMC4957450

[32] Selinger CP , Eaden J, Jones DB, et al Modifiable factors associated with nonadherence to maintenance medication for inflammatory bowel disease. Inflamm Bowel Dis. 2013;19(10):2199–2206. 23899547 10.1097/MIB.0b013e31829ed8a6

[33] Paes AH , Bakker A, Soe-Agnie CJ. Impact of dosage frequency on patient compliance. Diabetes Care. 1997;20(10):1512–1517. 9314626 10.2337/diacare.20.10.1512

[34] Feagan BG , Macdonald JK. Oral 5-aminosalicylic acid for induction of remission in ulcerative colitis. Cochrane Database Syst Rev. 2012;10:Cd000543. doi:10.1002/14651858.CD000543.pub3 23076889

[35] Pittet V , Rogler G, Mottet C, et al; Swiss IBD Cohort Study Group. Patients’ information-seeking activity is associated with treatment compliance in inflammatory bowel disease patients. Scand J Gastroenterol. 2014;49(6):662–673. 24617517 10.3109/00365521.2014.896408

[36] Berry AC , Dholaria K, Czul-Gurdian F, et al Relationship between patient knowledge and medication adherence in inflammatory bowel disease. Inflamm Bowel Dis. 2017;23(6):E39–E40. 28505053 10.1097/MIB.0000000000001156 PMC6093186

[37] Moradkhani A , Kerwin L, Dudley-Brown S, Tabibian JH. Disease-specific knowledge, coping, and adherence in patients with inflammatory bowel disease. Dig Dis Sci. 2011;56(10):2972–2977. 21538016 10.1007/s10620-011-1714-y

[38] Linn AJ , van Dijk L, Smit EG, et al May you never forget what is worth remembering: the relation between recall of medical information and medication adherence in patients with inflammatory bowel disease. J Crohns Colitis. 2013;7(11):e543–e550. 23660489 10.1016/j.crohns.2013.04.001

[39] Tiao DK , Chan W, Jeganathan J, et al Inflammatory bowel disease pharmacist adherence counseling improves medication adherence in Crohn’s disease and ulcerative colitis. Inflamm Bowel Dis. 2017;23(8):1257–1261. 28719539 10.1097/MIB.0000000000001194

[40] Waters BM , Jensen L, Fedorak RN. Effects of formal education for patients with inflammatory bowel disease: a randomized controlled trial. Can J Gastroenterol. 2005;19(4):235–244. 15861266 10.1155/2005/250504

[41] Bernal I , Domènech E, Garcia-Planella E, et al Medication-taking behavior in a cohort of patients with inflammatory bowel disease. Dig Dis Sci. 2006;51(12):2165–2169. 17086434 10.1007/s10620-006-9444-2

[42] Selinger CP , Robinson A, Leong RW. Clinical impact and drivers of non-adherence to maintenance medication for inflammatory bowel disease. Expert Opin Drug Saf. 2011;10(6):863–870. 21548837 10.1517/14740338.2011.583915

[43] Haynes RB , Ackloo E, Sahota N, McDonald HP, Yao X. Interventions for enhancing medication adherence. Cochrane Database Systematic Rev. 2008;(2):Cd000011. doi:10.1002/14651858.CD000011.pub3 18425859

