# Supplementary material for: Adherence to Inflammatory Bowel Disease Medications in Southern New Zealand
Source: Crohns Colitis 360. 2021 Aug 2;3(3):otab056. doi: 10.1093/crocol/otab056 (PMC9802163; doi:10.1093/crocol/otab056)
Supplement: otab056_suppl_Supplementary_Appendix [file otab056_suppl_supplementary_appendix.docx]

*Supplementary Figure 1: Use of medications in the study sample (total=144)*


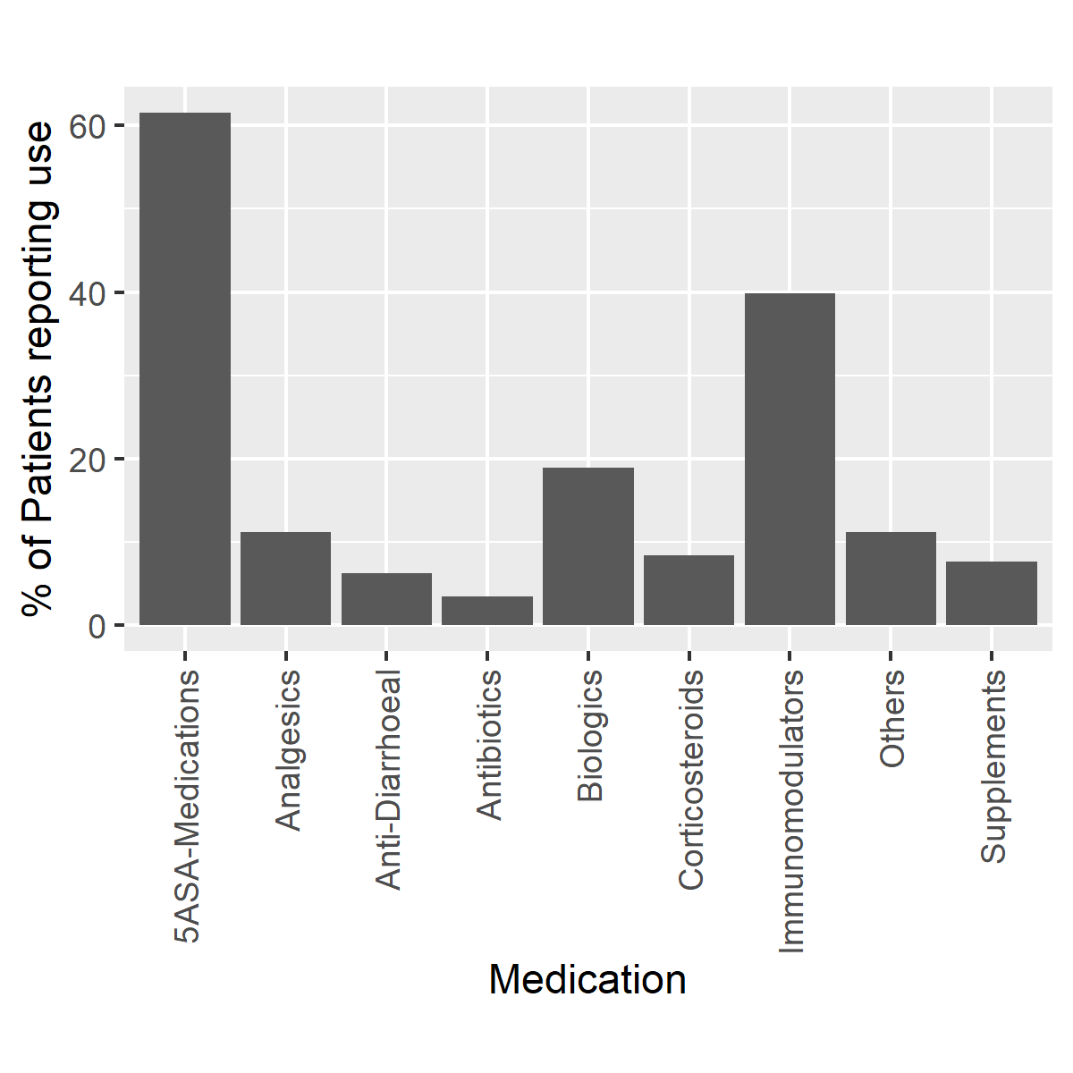


*Supplementary Figure 2: ProMAS medication adherence scores based on if the participants listed any specific circumstances for medication non-adherence*


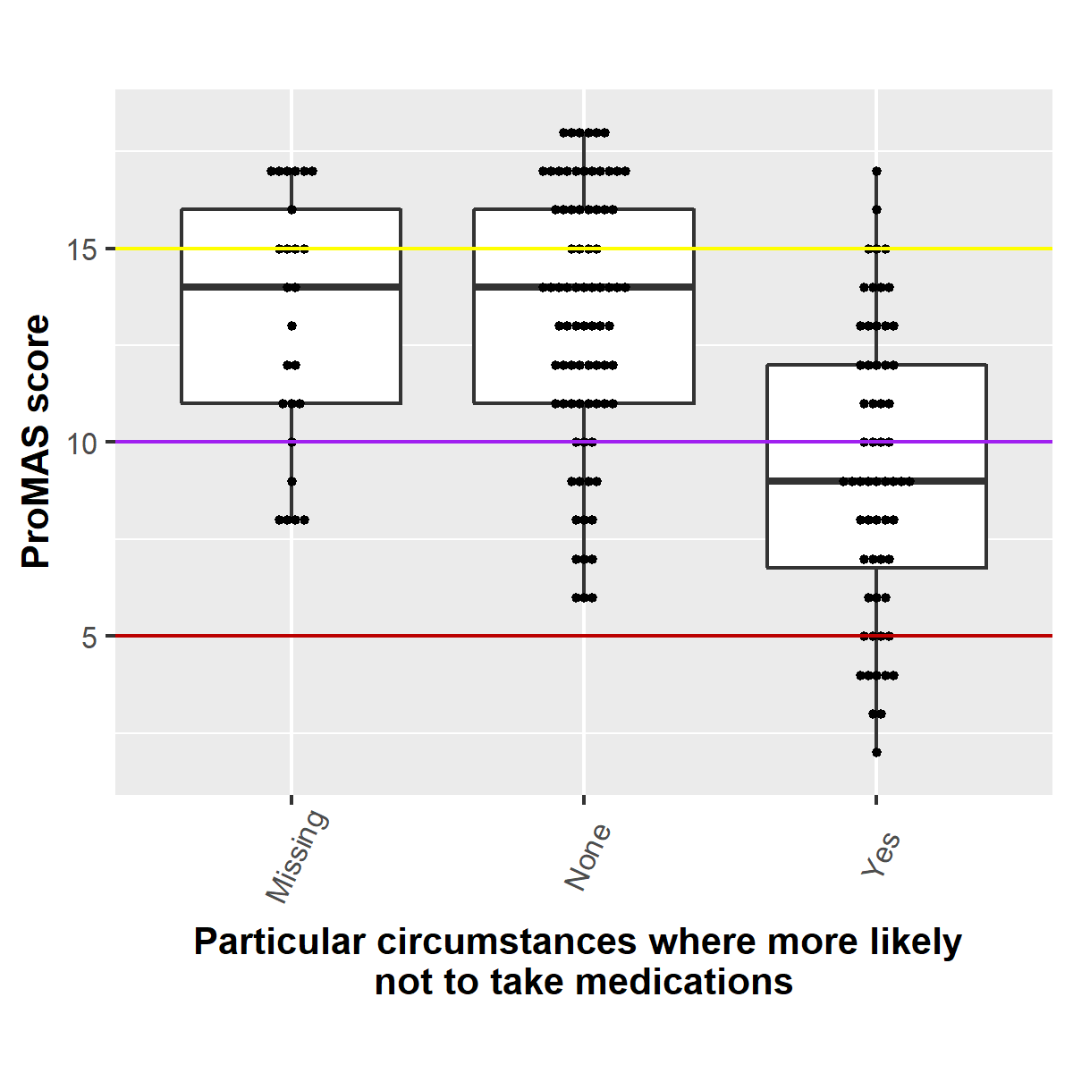


** The boxplots show median, 25^th^ and 75^th^ percentile of scores per age group along with distribution of all the scores; dark red line indicates low adherence, purple – low-medium adherence, yellow – high adherence*
